# Bis(μ-5-hydr­oxy-2-{[2-(*N*-phenyl­thio­carbamo­yl)hydrazin-1-yl­idene]meth­yl}phenolato)bis[chloridozinc(II)] *N*,*N*-dimethyl­formamide tetra­solvate

**DOI:** 10.1107/S1600536810014522

**Published:** 2010-04-24

**Authors:** Kong Wai Tan, Chew Hee Ng, M. Jamil Maah, Seik Weng Ng

**Affiliations:** a Department of Chemistry, University of Malaya, 50603 Kuala Lumpur, Malaysia; b Faculty of Engineering and Science, Universiti Tunku Abdul Rahman, 53300 Kuala Lumpur, Malaysia

## Abstract

In the dinuclear title compound, [Zn_2_(C_14_H_12_N_3_O_2_S)_2_Cl_2_]·4C_3_H_7_NO, the two monodeprotonated Schiff base ligands *N*,*O*,*S*:*O*-chelate to Zn atoms. The formally negatively charged O atom involved in chelation also serves as a bridge. The O, O′, N and S atoms comprise a square, and the Cl atom the apex of a square pyramid surrounding each metal atom. The solvate dimethyl­formamide mol­ecules, one of which is disordered over two positions in a 3:1 ratio, are hydrogen bonded to the dinuclear mol­ecule.

## Related literature

For related zinc complexes, see: Tan *et al.* (2009*a* ▶,*b* ▶).
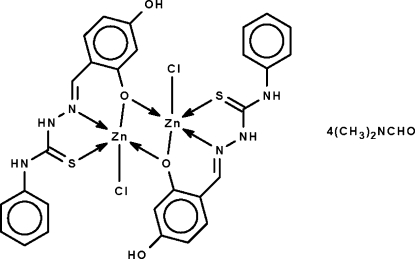


## Experimental

### 

#### Crystal data

[Zn_2_(C_14_H_12_N_3_O_2_S)_2_Cl_2_]·4C_3_H_7_NO
*M* *_r_* = 1066.68
Triclinic, 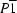

*a* = 8.1913 (4) Å
*b* = 17.6402 (7) Å
*c* = 17.9597 (7) Å
α = 64.941 (3)°
β = 81.213 (3)°
γ = 89.103 (3)°
*V* = 2319.76 (17) Å^3^
*Z* = 2
Mo *K*α radiation
μ = 1.30 mm^−1^
*T* = 100 K
0.12 × 0.08 × 0.04 mm

#### Data collection

Bruker SMART APEX diffractometer
Absorption correction: multi-scan (*SADABS*; Sheldrick, 1996 ▶) *T*_min_ = 0.860, *T*_max_ = 0.950
15848 measured reflections
8106 independent reflections
4918 reflections with *I* > 2σ(*I*)
*R*_int_ = 0.071

#### Refinement

*R*[*F* ^2^ > 2σ(*F* ^2^)] = 0.055
*wR*(*F* ^2^) = 0.161
*S* = 0.97
8106 reflections
634 parameters
68 restraints
H-atom parameters constrained
Δρ_max_ = 0.59 e Å^−3^
Δρ_min_ = −0.71 e Å^−3^

### 

Data collection: *APEX2* (Bruker, 2009 ▶); cell refinement: *SAINT* (Bruker, 2009 ▶); data reduction: *SAINT*; program(s) used to solve structure: *SHELXS97* (Sheldrick, 2008 ▶); program(s) used to refine structure: *SHELXL97* (Sheldrick, 2008 ▶); molecular graphics: *X-SEED* (Barbour, 2001 ▶); software used to prepare material for publication: *publCIF* (Westrip, 2010 ▶).

## Supplementary Material

Crystal structure: contains datablocks global, I. DOI: 10.1107/S1600536810014522/hg2675sup1.cif

Structure factors: contains datablocks I. DOI: 10.1107/S1600536810014522/hg2675Isup2.hkl

Additional supplementary materials:  crystallographic information; 3D view; checkCIF report

## Figures and Tables

**Table 1:** Selected bond lengths (Å)

Zn1—N1	2.127 (5)
Zn1—O1	2.073 (4)
Zn1—O3	2.024 (4)
Zn1—S1	2.464 (2)
Zn1—Cl1	2.272 (2)
Zn2—O1	2.024 (4)
Zn2—O3	2.061 (4)
Zn2—N4	2.116 (5)
Zn2—S2	2.420 (2)
Zn2—Cl2	2.264 (2)

**Table 2:** Hydrogen-bond geometry (Å, °)

*D*—H⋯*A*	*D*—H	H⋯*A*	*D*⋯*A*	*D*—H⋯*A*
O2—H2*o*⋯O5	0.84	1.76	2.595 (9)	170
O4—H4*o*⋯O6	0.84	1.82	2.657 (6)	175
N2—H2⋯O7	0.88	1.89	2.713 (6)	156
N3—H3⋯O7	0.84	2.18	2.940 (6)	151
N5—H5⋯O8	0.88	1.90	2.717 (6)	154
N6—H6⋯O8	0.84	2.08	2.841 (6)	150

**Fig. 1.**
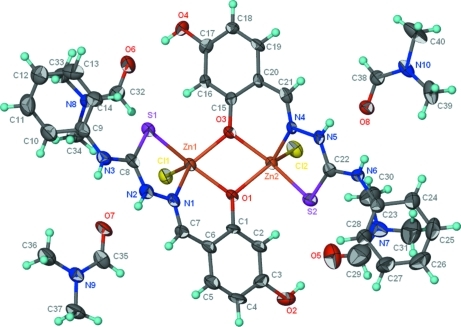
Thermal ellipsoid plot (Barbour, 2001) of Zn2Cl2(C14H12N3O2S)2.4DMF at the 70% probability level; hydrogen atoms are drawn as spheres of arbitrary radius. The disorder in one of the four DMF molecules is not shown.

## supplementary crystallographic information

### Experimental

Zinc chloride (0.14 g, 1 mmol) and 2,4-dihydroxybenzaldehyde 4-phenyl thiosemicarbazone (0.29 g, 1 mmol) were heated in ethanol (20 ml) for 3 hours. The compound that separated on cooling the solution was recrystallized from a mixture of ethanol and DMF.

### Refinement

Carbon-, nitrogen- and oxygen-bound H-atoms were placed in calculated positions (C—H 0.95 to 0.98, N–H 0.86, O–H 0.84 Å) and were included in the refinement in the riding model approximation, with *U*(H) set to 1.2 to 1.5*U*(*C*,*N*,*O*).

One of the four DMF molecules is disordered over two positions; as the disorder refined to a near 3:1 ratio, this is fixed as exactly 3:1. Pairs of distances were restrained to within 0.01 Å of each other. Each component was restrained to be nearly flat. The anisotropic temperature factors were restrained to be nearly isotropic.

### Crystal data

**Table:**

[Zn_2_(C_14_H_12_N_3_O_2_S)_2_Cl_2_]·4C_3_H_7_NO	*Z* = 2
*M**_r_* = 1066.68	*F*(000) = 1104
Triclinic, *P*1	*D*_x_ = 1.527 Mg m^−^^3^
Hall symbol: -P 1	Mo *K*α radiation, λ = 0.71073 Å
*a* = 8.1913 (4) Å	Cell parameters from 1271 reflections
*b* = 17.6402 (7) Å	θ = 2.3–19.6°
*c* = 17.9597 (7) Å	µ = 1.30 mm^−^^1^
α = 64.941 (3)°	*T* = 100 K
β = 81.213 (3)°	Prism, yellow
γ = 89.103 (3)°	0.12 × 0.08 × 0.04 mm
*V* = 2319.76 (17) Å^3^	

### Data collection

**Table:**

Bruker SMART APEX diffractometer	8106 independent reflections
Radiation source: fine-focus sealed tube	4918 reflections with *I* > 2σ(*I*)
graphite	*R*_int_ = 0.071
ω scans	θ_max_ = 25.0°, θ_min_ = 1.3°
Absorption correction: multi-scan (*SADABS*; Sheldrick, 1996)	*h* = −9→9
*T*_min_ = 0.860, *T*_max_ = 0.950	*k* = −20→20
15848 measured reflections	*l* = −20→21

### Refinement

**Table:**

Refinement on *F*^2^	Primary atom site location: structure-invariant direct methods
Least-squares matrix: full	Secondary atom site location: difference Fourier map
*R*[*F*^2^ > 2σ(*F*^2^)] = 0.055	Hydrogen site location: inferred from neighbouring sites
*wR*(*F*^2^) = 0.161	H-atom parameters constrained
*S* = 0.97	*w* = 1/[σ^2^(*F*_o_^2^) + (0.0745*P*)^2^] where *P* = (*F*_o_^2^ + 2*F*_c_^2^)/3
8106 reflections	(Δ/σ)_max_ = 0.001
634 parameters	Δρ_max_ = 0.59 e Å^−^^3^
68 restraints	Δρ_min_ = −0.70 e Å^−^^3^

### Fractional atomic coordinates and isotropic or equivalent isotropic displacement parameters (Å^2^)

**Table:**

	*x*	*y*	*z*	*U*_iso_*/*U*_eq_	Occ. (<1)
Zn1	0.41630 (9)	0.73410 (4)	0.85750 (4)	0.0238 (2)	
Zn2	0.50793 (9)	0.77020 (4)	0.66356 (4)	0.0224 (2)	
Cl1	0.16292 (19)	0.66906 (9)	0.92067 (9)	0.0294 (4)	
Cl2	0.2909 (2)	0.78623 (10)	0.59478 (10)	0.0332 (4)	
S1	0.4684 (2)	0.81633 (9)	0.93413 (9)	0.0273 (4)	
S2	0.7294 (2)	0.71361 (9)	0.60021 (10)	0.0292 (4)	
O1	0.4913 (5)	0.6764 (2)	0.7795 (2)	0.0245 (10)	
O2	0.5357 (6)	0.4359 (3)	0.7225 (3)	0.0408 (12)	
H2o	0.5036	0.4694	0.6785	0.061*	
O3	0.4394 (5)	0.8279 (2)	0.7412 (2)	0.0239 (9)	
O4	0.1114 (5)	1.0395 (2)	0.7781 (3)	0.0294 (10)	
H4o	0.0849	1.0010	0.8262	0.044*	
O6	0.0090 (6)	0.9223 (3)	0.9310 (3)	0.0423 (12)	
O7	0.8112 (5)	0.5952 (3)	1.1131 (3)	0.0360 (11)	
O8	1.0048 (5)	0.9906 (2)	0.4063 (2)	0.0288 (10)	
N1	0.5986 (6)	0.6606 (3)	0.9244 (3)	0.0260 (12)	
N2	0.6490 (6)	0.6813 (3)	0.9837 (3)	0.0293 (13)	
H2	0.7204	0.6506	1.0143	0.035*	
N3	0.6376 (7)	0.7521 (3)	1.0620 (3)	0.0319 (13)	
H3	0.6976	0.7143	1.0885	0.038*	
N4	0.6596 (6)	0.8812 (3)	0.5933 (3)	0.0196 (11)	
N5	0.7916 (6)	0.8787 (3)	0.5369 (3)	0.0231 (12)	
H5	0.8501	0.9251	0.5024	0.028*	
N6	0.9575 (6)	0.8135 (3)	0.4732 (3)	0.0244 (12)	
H6	1.0063	0.8612	0.4472	0.029*	
N8	0.0525 (7)	0.8249 (3)	1.0570 (3)	0.0289 (13)	
N9	0.9143 (6)	0.4839 (3)	1.2119 (3)	0.0286 (12)	
N10	1.0845 (6)	1.1074 (3)	0.2867 (3)	0.0319 (13)	
C1	0.5341 (7)	0.5977 (3)	0.8007 (4)	0.0225 (14)	
C2	0.5085 (7)	0.5574 (3)	0.7506 (4)	0.0268 (15)	
H2A	0.4578	0.5859	0.7027	0.032*	
C3	0.5561 (8)	0.4766 (4)	0.7704 (4)	0.0313 (16)	
C4	0.6296 (8)	0.4348 (4)	0.8402 (4)	0.0350 (17)	
H4A	0.6638	0.3796	0.8531	0.042*	
C5	0.6531 (8)	0.4718 (4)	0.8898 (4)	0.0337 (16)	
H5A	0.7016	0.4415	0.9382	0.040*	
C6	0.6075 (7)	0.5545 (4)	0.8720 (4)	0.0268 (15)	
C7	0.6436 (8)	0.5892 (4)	0.9270 (4)	0.0277 (15)	
H7A	0.7062	0.5569	0.9691	0.033*	
C8	0.5905 (8)	0.7475 (4)	0.9951 (4)	0.0263 (15)	
C9	0.5975 (8)	0.8134 (4)	1.0929 (4)	0.0300 (15)	
C10	0.5199 (10)	0.7877 (4)	1.1740 (4)	0.045 (2)	
H10	0.4901	0.7301	1.2082	0.054*	
C11	0.4855 (11)	0.8470 (5)	1.2053 (5)	0.056 (2)	
H11	0.4320	0.8296	1.2612	0.067*	
C12	0.5281 (9)	0.9301 (5)	1.1565 (4)	0.0412 (18)	
H12	0.5037	0.9700	1.1787	0.049*	
C13	0.6059 (9)	0.9564 (4)	1.0753 (5)	0.0412 (18)	
H13	0.6355	1.0141	1.0416	0.049*	
C14	0.6406 (8)	0.8978 (4)	1.0435 (4)	0.0339 (16)	
H14	0.6941	0.9155	0.9875	0.041*	
C15	0.3996 (7)	0.9082 (3)	0.7144 (3)	0.0207 (13)	
C16	0.2762 (7)	0.9312 (3)	0.7617 (3)	0.0203 (13)	
H16	0.2196	0.8899	0.8128	0.024*	
C17	0.2344 (7)	1.0147 (4)	0.7347 (4)	0.0239 (14)	
C18	0.3160 (8)	1.0763 (4)	0.6607 (4)	0.0261 (15)	
H18	0.2871	1.1330	0.6427	0.031*	
C19	0.4385 (7)	1.0544 (3)	0.6138 (4)	0.0240 (14)	
H19	0.4952	1.0969	0.5635	0.029*	
C20	0.4839 (7)	0.9705 (3)	0.6375 (3)	0.0207 (13)	
C21	0.6179 (7)	0.9557 (4)	0.5841 (4)	0.0250 (14)	
H21	0.6789	1.0027	0.5398	0.030*	
C22	0.8311 (7)	0.8049 (4)	0.5348 (4)	0.0231 (14)	
C23	1.0165 (7)	0.7507 (4)	0.4479 (4)	0.0273 (15)	
C24	1.0604 (8)	0.7757 (4)	0.3625 (4)	0.0389 (18)	
H24	1.0491	0.8319	0.3243	0.047*	
C25	1.1209 (10)	0.7171 (5)	0.3341 (5)	0.058 (2)	
H25	1.1517	0.7342	0.2759	0.069*	
C26	1.1372 (10)	0.6363 (5)	0.3869 (5)	0.054 (2)	
H26	1.1789	0.5972	0.3662	0.065*	
C27	1.0927 (10)	0.6124 (5)	0.4704 (5)	0.055 (2)	
H27	1.1014	0.5556	0.5079	0.066*	
C28	1.0351 (9)	0.6691 (4)	0.5015 (4)	0.045 (2)	
H28	1.0085	0.6515	0.5600	0.054*	
C32	0.0373 (8)	0.8497 (4)	0.9781 (4)	0.0338 (16)	
H32	0.0487	0.8094	0.9558	0.041*	
C33	0.0466 (10)	0.8813 (4)	1.0970 (4)	0.0449 (19)	
H33A	0.0283	0.9380	1.0569	0.067*	
H33B	0.1516	0.8815	1.1168	0.067*	
H33C	−0.0441	0.8624	1.1443	0.067*	
C34	0.0917 (10)	0.7388 (4)	1.1072 (4)	0.045 (2)	
H34A	0.1997	0.7389	1.1242	0.067*	
H34B	0.0949	0.7064	1.0742	0.067*	
H34C	0.0067	0.7135	1.1567	0.067*	
C35	0.8629 (8)	0.5233 (4)	1.1402 (4)	0.0337 (16)	
H35	0.8653	0.4944	1.1058	0.040*	
C36	0.9132 (11)	0.5215 (4)	1.2697 (4)	0.054 (2)	
H36A	0.8704	0.5776	1.2459	0.081*	
H36B	1.0261	0.5258	1.2799	0.081*	
H36C	0.8423	0.4866	1.3223	0.081*	
C37	0.9689 (9)	0.3989 (4)	1.2394 (4)	0.0415 (18)	
H37A	0.9797	0.3825	1.1932	0.062*	
H37B	0.8877	0.3606	1.2858	0.062*	
H37C	1.0763	0.3963	1.2579	0.062*	
C38	0.9962 (8)	1.0668 (4)	0.3608 (4)	0.0267 (15)	
H38	0.9206	1.0975	0.3814	0.032*	
C39	1.1953 (8)	1.0671 (5)	0.2467 (4)	0.0430 (19)	
H39A	1.3079	1.0916	0.2356	0.065*	
H39B	1.1608	1.0751	0.1941	0.065*	
H39C	1.1929	1.0070	0.2831	0.065*	
C40	1.0718 (10)	1.1982 (4)	0.2402 (5)	0.055 (2)	
H40A	0.9887	1.2181	0.2721	0.083*	
H40B	1.0389	1.2098	0.1863	0.083*	
H40C	1.1792	1.2270	0.2311	0.083*	
O5	0.4714 (11)	0.5477 (6)	0.5814 (5)	0.060 (2)	0.75
N7	0.5710 (9)	0.6399 (6)	0.4550 (7)	0.039 (2)	0.75
C29	0.5636 (14)	0.5685 (8)	0.5180 (8)	0.071 (4)	0.75
H29	0.6391	0.5288	0.5133	0.085*	0.75
C30	0.4685 (14)	0.7083 (6)	0.4500 (7)	0.057 (3)	0.75
H30A	0.3866	0.6907	0.5009	0.086*	0.75
H30B	0.4115	0.7246	0.4017	0.086*	0.75
H30C	0.5376	0.7560	0.4440	0.086*	0.75
C31	0.6916 (14)	0.6569 (8)	0.3799 (7)	0.069 (3)	0.75
H31A	0.7673	0.6112	0.3911	0.103*	0.75
H31B	0.7548	0.7097	0.3638	0.103*	0.75
H31C	0.6335	0.6612	0.3346	0.103*	0.75
O5'	0.379 (3)	0.5363 (15)	0.5975 (14)	0.053 (7)	0.25
N7'	0.554 (3)	0.613 (2)	0.493 (2)	0.085 (18)	0.25
C29'	0.412 (3)	0.6011 (14)	0.5386 (14)	0.043 (7)	0.25
H29'	0.3345	0.6434	0.5253	0.052*	0.25
C30'	0.591 (5)	0.693 (2)	0.422 (2)	0.075 (13)	0.25
H30D	0.5722	0.6868	0.3719	0.112*	0.25
H30E	0.7066	0.7107	0.4155	0.112*	0.25
H30F	0.5186	0.7344	0.4297	0.112*	0.25
C31'	0.699 (4)	0.5632 (18)	0.4958 (19)	0.050 (8)	0.25
H31D	0.6913	0.5165	0.5512	0.074*	0.25
H31E	0.7993	0.5983	0.4840	0.074*	0.25
H31F	0.7024	0.5410	0.4539	0.074*	0.25

### Atomic displacement parameters (Å^2^)

**Table:**

	*U*^11^	*U*^22^	*U*^33^	*U*^12^	*U*^13^	*U*^23^
Zn1	0.0287 (4)	0.0219 (4)	0.0169 (4)	0.0053 (3)	−0.0028 (3)	−0.0048 (3)
Zn2	0.0261 (4)	0.0203 (4)	0.0175 (4)	0.0028 (3)	−0.0025 (3)	−0.0051 (3)
Cl1	0.0299 (9)	0.0307 (9)	0.0230 (8)	0.0019 (7)	−0.0032 (7)	−0.0074 (7)
Cl2	0.0311 (9)	0.0346 (9)	0.0289 (9)	0.0010 (7)	−0.0090 (7)	−0.0072 (7)
S1	0.0324 (9)	0.0255 (9)	0.0215 (8)	0.0047 (7)	−0.0063 (7)	−0.0071 (7)
S2	0.0323 (9)	0.0212 (8)	0.0273 (9)	0.0030 (7)	0.0017 (7)	−0.0061 (7)
O1	0.034 (3)	0.017 (2)	0.017 (2)	0.0053 (18)	−0.0033 (19)	−0.0023 (18)
O2	0.058 (3)	0.024 (3)	0.042 (3)	0.010 (2)	−0.013 (3)	−0.014 (2)
O3	0.030 (2)	0.019 (2)	0.018 (2)	0.0033 (18)	−0.0009 (19)	−0.0038 (18)
O4	0.030 (2)	0.031 (3)	0.026 (2)	0.009 (2)	0.003 (2)	−0.014 (2)
O6	0.052 (3)	0.042 (3)	0.025 (3)	0.016 (2)	−0.004 (2)	−0.008 (2)
O7	0.045 (3)	0.025 (3)	0.026 (2)	0.006 (2)	−0.010 (2)	0.001 (2)
O8	0.031 (3)	0.024 (2)	0.026 (2)	0.0010 (18)	−0.001 (2)	−0.006 (2)
N1	0.031 (3)	0.025 (3)	0.017 (3)	0.004 (2)	−0.006 (2)	−0.003 (2)
N2	0.033 (3)	0.030 (3)	0.025 (3)	0.009 (2)	−0.011 (3)	−0.010 (3)
N3	0.046 (4)	0.026 (3)	0.024 (3)	0.008 (3)	−0.015 (3)	−0.008 (2)
N4	0.020 (3)	0.022 (3)	0.013 (2)	0.006 (2)	−0.001 (2)	−0.005 (2)
N5	0.024 (3)	0.025 (3)	0.016 (3)	0.001 (2)	0.004 (2)	−0.006 (2)
N6	0.027 (3)	0.018 (3)	0.022 (3)	0.001 (2)	0.005 (2)	−0.006 (2)
N8	0.043 (3)	0.023 (3)	0.019 (3)	0.007 (2)	−0.002 (3)	−0.009 (2)
N9	0.031 (3)	0.029 (3)	0.025 (3)	0.004 (2)	−0.012 (3)	−0.008 (3)
N10	0.028 (3)	0.030 (3)	0.024 (3)	−0.005 (2)	−0.004 (3)	0.001 (3)
C1	0.024 (3)	0.012 (3)	0.023 (3)	0.005 (2)	0.003 (3)	−0.002 (3)
C2	0.029 (4)	0.016 (3)	0.026 (3)	0.003 (3)	0.000 (3)	−0.001 (3)
C3	0.033 (4)	0.028 (4)	0.035 (4)	0.005 (3)	0.002 (3)	−0.017 (3)
C4	0.042 (4)	0.015 (3)	0.037 (4)	0.008 (3)	−0.004 (3)	−0.001 (3)
C5	0.038 (4)	0.024 (4)	0.028 (4)	0.001 (3)	0.001 (3)	−0.002 (3)
C6	0.028 (4)	0.019 (3)	0.023 (3)	0.001 (3)	0.001 (3)	0.000 (3)
C7	0.031 (4)	0.025 (4)	0.023 (3)	0.011 (3)	−0.005 (3)	−0.006 (3)
C8	0.030 (4)	0.022 (3)	0.020 (3)	−0.001 (3)	−0.005 (3)	−0.002 (3)
C9	0.037 (4)	0.029 (4)	0.025 (4)	0.001 (3)	−0.017 (3)	−0.009 (3)
C10	0.074 (6)	0.030 (4)	0.030 (4)	−0.007 (4)	−0.013 (4)	−0.009 (3)
C11	0.089 (7)	0.051 (5)	0.031 (4)	−0.014 (5)	−0.008 (4)	−0.021 (4)
C12	0.048 (5)	0.043 (5)	0.038 (4)	0.001 (4)	−0.016 (4)	−0.020 (4)
C13	0.041 (4)	0.031 (4)	0.051 (5)	0.003 (3)	−0.015 (4)	−0.014 (4)
C14	0.039 (4)	0.028 (4)	0.032 (4)	0.003 (3)	−0.008 (3)	−0.010 (3)
C15	0.025 (3)	0.024 (3)	0.017 (3)	0.005 (3)	−0.011 (3)	−0.011 (3)
C16	0.022 (3)	0.022 (3)	0.015 (3)	0.000 (2)	−0.003 (3)	−0.007 (3)
C17	0.017 (3)	0.033 (4)	0.027 (3)	0.010 (3)	−0.010 (3)	−0.017 (3)
C18	0.037 (4)	0.022 (3)	0.021 (3)	0.012 (3)	−0.012 (3)	−0.009 (3)
C19	0.027 (3)	0.019 (3)	0.019 (3)	0.003 (3)	−0.006 (3)	0.000 (3)
C20	0.021 (3)	0.025 (3)	0.013 (3)	0.005 (2)	−0.004 (3)	−0.006 (3)
C21	0.024 (3)	0.029 (4)	0.018 (3)	−0.001 (3)	−0.005 (3)	−0.005 (3)
C22	0.024 (3)	0.026 (3)	0.021 (3)	0.005 (3)	−0.010 (3)	−0.009 (3)
C23	0.026 (4)	0.029 (4)	0.026 (4)	0.004 (3)	−0.004 (3)	−0.012 (3)
C24	0.044 (4)	0.042 (4)	0.030 (4)	0.014 (3)	−0.007 (3)	−0.015 (3)
C25	0.071 (6)	0.072 (6)	0.034 (4)	0.018 (5)	−0.001 (4)	−0.030 (5)
C26	0.055 (5)	0.048 (5)	0.067 (6)	0.012 (4)	0.001 (5)	−0.037 (5)
C27	0.072 (6)	0.035 (4)	0.050 (5)	0.012 (4)	0.012 (4)	−0.018 (4)
C28	0.059 (5)	0.038 (4)	0.032 (4)	0.019 (4)	0.002 (4)	−0.013 (4)
C32	0.030 (4)	0.035 (4)	0.033 (4)	0.009 (3)	0.002 (3)	−0.014 (3)
C33	0.069 (5)	0.032 (4)	0.035 (4)	0.016 (4)	−0.013 (4)	−0.015 (3)
C34	0.073 (6)	0.023 (4)	0.035 (4)	0.009 (4)	−0.009 (4)	−0.011 (3)
C35	0.027 (4)	0.039 (4)	0.029 (4)	0.000 (3)	−0.009 (3)	−0.007 (3)
C36	0.089 (7)	0.039 (5)	0.034 (4)	0.013 (4)	−0.024 (4)	−0.010 (4)
C37	0.046 (5)	0.029 (4)	0.038 (4)	0.009 (3)	−0.012 (4)	−0.002 (3)
C38	0.028 (4)	0.027 (4)	0.026 (4)	−0.001 (3)	−0.006 (3)	−0.012 (3)
C39	0.038 (4)	0.064 (5)	0.025 (4)	−0.007 (4)	−0.009 (3)	−0.015 (4)
C40	0.051 (5)	0.042 (5)	0.047 (5)	−0.003 (4)	−0.020 (4)	0.010 (4)
O5	0.067 (6)	0.074 (5)	0.037 (4)	−0.003 (5)	−0.010 (4)	−0.021 (4)
N7	0.047 (5)	0.028 (5)	0.044 (5)	−0.004 (4)	−0.020 (4)	−0.012 (4)
C29	0.084 (8)	0.065 (8)	0.077 (8)	0.002 (6)	−0.018 (6)	−0.041 (6)
C30	0.059 (6)	0.058 (6)	0.060 (6)	0.021 (5)	−0.009 (5)	−0.031 (5)
C31	0.076 (7)	0.084 (7)	0.060 (6)	−0.002 (6)	−0.013 (6)	−0.043 (6)
O5'	0.054 (11)	0.057 (10)	0.051 (11)	0.001 (8)	−0.020 (8)	−0.020 (8)
N7'	0.10 (2)	0.08 (2)	0.09 (2)	−0.016 (9)	−0.018 (10)	−0.046 (12)
C29'	0.053 (11)	0.041 (11)	0.045 (11)	0.008 (8)	−0.008 (9)	−0.027 (8)
C30'	0.070 (15)	0.081 (15)	0.077 (15)	0.001 (10)	−0.012 (9)	−0.035 (10)
C31'	0.049 (11)	0.049 (11)	0.052 (11)	0.002 (9)	−0.012 (9)	−0.022 (9)

### Geometric parameters (Å, °)

**Table:**

Zn1—N1	2.127 (5)	C15—C16	1.388 (7)
Zn1—O1	2.073 (4)	C15—C20	1.427 (8)
Zn1—O3	2.024 (4)	C16—C17	1.396 (8)
Zn1—S1	2.464 (2)	C16—H16	0.9500
Zn1—Cl1	2.272 (2)	C17—C18	1.387 (8)
Zn2—O1	2.024 (4)	C18—C19	1.369 (8)
Zn2—O3	2.061 (4)	C18—H18	0.9500
Zn2—N4	2.116 (5)	C19—C20	1.416 (8)
Zn2—S2	2.420 (2)	C19—H19	0.9500
Zn2—Cl2	2.264 (2)	C20—C21	1.441 (8)
S1—C8	1.686 (6)	C21—H21	0.9500
S2—C22	1.675 (6)	C23—C28	1.375 (9)
O1—C1	1.332 (6)	C23—C24	1.394 (9)
O2—C3	1.360 (7)	C24—C25	1.387 (9)
O2—H2o	0.8400	C24—H24	0.9500
O3—C15	1.341 (6)	C25—C26	1.356 (10)
O4—C17	1.359 (7)	C25—H25	0.9500
O4—H4o	0.8400	C26—C27	1.363 (10)
O6—C32	1.241 (7)	C26—H26	0.9500
O7—C35	1.245 (7)	C27—C28	1.383 (9)
O8—C38	1.250 (7)	C27—H27	0.9500
N1—C7	1.290 (7)	C28—H28	0.9500
N1—N2	1.380 (7)	C32—H32	0.9500
N2—C8	1.339 (7)	C33—H33A	0.9800
N2—H2	0.8800	C33—H33B	0.9800
N3—C8	1.352 (8)	C33—H33C	0.9800
N3—C9	1.423 (8)	C34—H34A	0.9800
N3—H3	0.8400	C34—H34B	0.9800
N4—C21	1.299 (7)	C34—H34C	0.9800
N4—N5	1.377 (6)	C35—H35	0.9500
N5—C22	1.351 (7)	C36—H36A	0.9800
N5—H5	0.8800	C36—H36B	0.9800
N6—C22	1.354 (7)	C36—H36C	0.9800
N6—C23	1.417 (7)	C37—H37A	0.9800
N6—H6	0.8400	C37—H37B	0.9800
N8—C32	1.320 (8)	C37—H37C	0.9800
N8—C33	1.450 (8)	C38—H38	0.9500
N8—C34	1.461 (7)	C39—H39A	0.9800
N9—C35	1.311 (8)	C39—H39B	0.9800
N9—C36	1.449 (8)	C39—H39C	0.9800
N9—C37	1.452 (7)	C40—H40A	0.9800
N10—C38	1.313 (7)	C40—H40B	0.9800
N10—C39	1.434 (8)	C40—H40C	0.9800
N10—C40	1.470 (8)	O5—C29	1.180 (14)
C1—C2	1.400 (8)	N7—C29	1.282 (18)
C1—C6	1.406 (8)	N7—C30	1.437 (11)
C2—C3	1.381 (8)	N7—C31	1.465 (14)
C2—H2A	0.9500	C29—H29	0.9500
C3—C4	1.382 (9)	C30—H30A	0.9800
C4—C5	1.344 (9)	C30—H30B	0.9800
C4—H4A	0.9500	C30—H30C	0.9800
C5—C6	1.413 (8)	C31—H31A	0.9800
C5—H5A	0.9500	C31—H31B	0.9800
C6—C7	1.431 (9)	C31—H31C	0.9800
C7—H7A	0.9500	O5'—C29'	1.182 (16)
C9—C10	1.378 (9)	N7'—C29'	1.27 (2)
C9—C14	1.391 (8)	N7'—C30'	1.437 (15)
C10—C11	1.388 (9)	N7'—C31'	1.464 (16)
C10—H10	0.9500	C29'—H29'	0.9500
C11—C12	1.369 (10)	C30'—H30D	0.9800
C11—H11	0.9500	C30'—H30E	0.9800
C12—C13	1.378 (10)	C30'—H30F	0.9800
C12—H12	0.9500	C31'—H31D	0.9800
C13—C14	1.385 (9)	C31'—H31E	0.9800
C13—H13	0.9500	C31'—H31F	0.9800
C14—H14	0.9500		
			
O3—Zn1—O1	75.50 (14)	O4—C17—C18	116.8 (5)
O3—Zn1—N1	130.41 (17)	O4—C17—C16	122.4 (5)
O1—Zn1—N1	82.67 (17)	C18—C17—C16	120.8 (5)
O3—Zn1—Cl1	118.76 (12)	C19—C18—C17	119.1 (5)
O1—Zn1—Cl1	101.42 (12)	C19—C18—H18	120.4
N1—Zn1—Cl1	108.86 (14)	C17—C18—H18	120.4
O3—Zn1—S1	99.12 (12)	C18—C19—C20	122.3 (5)
O1—Zn1—S1	152.95 (13)	C18—C19—H19	118.8
N1—Zn1—S1	81.11 (14)	C20—C19—H19	118.8
Cl1—Zn1—S1	104.25 (6)	C19—C20—C15	117.6 (5)
O1—Zn2—O3	75.80 (15)	C19—C20—C21	117.1 (5)
O1—Zn2—N4	135.26 (17)	C15—C20—C21	125.2 (5)
O3—Zn2—N4	83.09 (16)	N4—C21—C20	123.3 (5)
O1—Zn2—Cl2	118.39 (13)	N4—C21—H21	118.3
O3—Zn2—Cl2	103.72 (12)	C20—C21—H21	118.3
N4—Zn2—Cl2	104.65 (13)	N5—C22—N6	112.7 (5)
O1—Zn2—S2	95.77 (11)	N5—C22—S2	123.0 (4)
O3—Zn2—S2	146.98 (13)	N6—C22—S2	124.3 (4)
N4—Zn2—S2	81.05 (12)	C28—C23—C24	119.1 (6)
Cl2—Zn2—S2	108.23 (6)	C28—C23—N6	124.6 (6)
C8—S1—Zn1	96.0 (2)	C24—C23—N6	116.4 (5)
C22—S2—Zn2	97.4 (2)	C25—C24—C23	118.9 (7)
C1—O1—Zn2	127.7 (4)	C25—C24—H24	120.5
C1—O1—Zn1	127.9 (4)	C23—C24—H24	120.5
Zn2—O1—Zn1	104.11 (16)	C26—C25—C24	122.0 (7)
C3—O2—H2o	109.5	C26—C25—H25	119.0
C15—O3—Zn1	131.1 (3)	C24—C25—H25	119.0
C15—O3—Zn2	123.7 (3)	C25—C26—C27	118.6 (7)
Zn1—O3—Zn2	104.55 (16)	C25—C26—H26	120.7
C17—O4—H4o	109.5	C27—C26—H26	120.7
C7—N1—N2	115.3 (5)	C26—C27—C28	121.4 (7)
C7—N1—Zn1	126.0 (4)	C26—C27—H27	119.3
N2—N1—Zn1	117.2 (4)	C28—C27—H27	119.3
C8—N2—N1	120.7 (5)	C23—C28—C27	120.0 (7)
C8—N2—H2	119.6	C23—C28—H28	120.0
N1—N2—H2	119.6	C27—C28—H28	120.0
C8—N3—C9	127.7 (5)	O6—C32—N8	124.4 (6)
C8—N3—H3	116.2	O6—C32—H32	117.8
C9—N3—H3	116.2	N8—C32—H32	117.8
C21—N4—N5	115.5 (5)	N8—C33—H33A	109.5
C21—N4—Zn2	124.8 (4)	N8—C33—H33B	109.5
N5—N4—Zn2	117.6 (3)	H33A—C33—H33B	109.5
C22—N5—N4	119.8 (5)	N8—C33—H33C	109.5
C22—N5—H5	120.1	H33A—C33—H33C	109.5
N4—N5—H5	120.1	H33B—C33—H33C	109.5
C22—N6—C23	127.0 (5)	N8—C34—H34A	109.5
C22—N6—H6	116.5	N8—C34—H34B	109.5
C23—N6—H6	116.5	H34A—C34—H34B	109.5
C32—N8—C33	123.1 (5)	N8—C34—H34C	109.5
C32—N8—C34	120.8 (5)	H34A—C34—H34C	109.5
C33—N8—C34	115.9 (5)	H34B—C34—H34C	109.5
C35—N9—C36	121.6 (6)	O7—C35—N9	125.8 (7)
C35—N9—C37	122.0 (6)	O7—C35—H35	117.1
C36—N9—C37	116.3 (5)	N9—C35—H35	117.1
C38—N10—C39	123.0 (6)	N9—C36—H36A	109.5
C38—N10—C40	120.2 (6)	N9—C36—H36B	109.5
C39—N10—C40	116.7 (6)	H36A—C36—H36B	109.5
O1—C1—C2	119.8 (5)	N9—C36—H36C	109.5
O1—C1—C6	121.0 (5)	H36A—C36—H36C	109.5
C2—C1—C6	119.2 (5)	H36B—C36—H36C	109.5
C3—C2—C1	120.6 (6)	N9—C37—H37A	109.5
C3—C2—H2A	119.7	N9—C37—H37B	109.5
C1—C2—H2A	119.7	H37A—C37—H37B	109.5
O2—C3—C2	122.2 (6)	N9—C37—H37C	109.5
O2—C3—C4	117.8 (6)	H37A—C37—H37C	109.5
C2—C3—C4	119.9 (6)	H37B—C37—H37C	109.5
C5—C4—C3	120.6 (6)	O8—C38—N10	124.5 (6)
C5—C4—H4A	119.7	O8—C38—H38	117.7
C3—C4—H4A	119.7	N10—C38—H38	117.7
C4—C5—C6	121.7 (6)	N10—C39—H39A	109.5
C4—C5—H5A	119.2	N10—C39—H39B	109.5
C6—C5—H5A	119.2	H39A—C39—H39B	109.5
C1—C6—C5	118.1 (6)	N10—C39—H39C	109.5
C1—C6—C7	124.6 (5)	H39A—C39—H39C	109.5
C5—C6—C7	117.3 (6)	H39B—C39—H39C	109.5
N1—C7—C6	125.6 (6)	N10—C40—H40A	109.5
N1—C7—H7A	117.2	N10—C40—H40B	109.5
C6—C7—H7A	117.2	H40A—C40—H40B	109.5
N2—C8—N3	112.3 (5)	N10—C40—H40C	109.5
N2—C8—S1	123.9 (5)	H40A—C40—H40C	109.5
N3—C8—S1	123.7 (5)	H40B—C40—H40C	109.5
C10—C9—C14	119.9 (6)	C29—N7—C30	125.6 (12)
C10—C9—N3	119.1 (6)	C29—N7—C31	119.9 (10)
C14—C9—N3	120.9 (6)	C30—N7—C31	114.5 (11)
C9—C10—C11	119.3 (6)	O5—C29—N7	125.9 (14)
C9—C10—H10	120.3	O5—C29—H29	117.1
C11—C10—H10	120.3	N7—C29—H29	117.1
C12—C11—C10	120.7 (7)	C29'—N7'—C30'	118 (3)
C12—C11—H11	119.7	C29'—N7'—C31'	134 (4)
C10—C11—H11	119.7	C30'—N7'—C31'	108 (3)
C11—C12—C13	120.5 (7)	O5'—C29'—N7'	119 (3)
C11—C12—H12	119.8	O5'—C29'—H29'	120.5
C13—C12—H12	119.8	N7'—C29'—H29'	120.5
C12—C13—C14	119.4 (7)	N7'—C30'—H30D	109.5
C12—C13—H13	120.3	N7'—C30'—H30E	109.5
C14—C13—H13	120.3	H30D—C30'—H30E	109.5
C13—C14—C9	120.3 (6)	N7'—C30'—H30F	109.5
C13—C14—H14	119.9	H30D—C30'—H30F	109.5
C9—C14—H14	119.9	H30E—C30'—H30F	109.5
O3—C15—C16	119.9 (5)	N7'—C31'—H31D	109.5
O3—C15—C20	120.5 (5)	N7'—C31'—H31E	109.5
C16—C15—C20	119.5 (5)	H31D—C31'—H31E	109.5
C15—C16—C17	120.5 (5)	N7'—C31'—H31F	109.5
C15—C16—H16	119.7	H31D—C31'—H31F	109.5
C17—C16—H16	119.7	H31E—C31'—H31F	109.5
			
O3—Zn1—S1—C8	−137.6 (2)	C2—C1—C6—C5	0.2 (9)
O1—Zn1—S1—C8	−61.6 (3)	O1—C1—C6—C7	−0.3 (9)
N1—Zn1—S1—C8	−7.8 (2)	C2—C1—C6—C7	178.6 (6)
Cl1—Zn1—S1—C8	99.5 (2)	C4—C5—C6—C1	0.8 (9)
O1—Zn2—S2—C22	−143.0 (2)	C4—C5—C6—C7	−177.7 (6)
O3—Zn2—S2—C22	−70.3 (3)	N2—N1—C7—C6	−179.5 (5)
N4—Zn2—S2—C22	−8.0 (2)	Zn1—N1—C7—C6	14.6 (9)
Cl2—Zn2—S2—C22	94.6 (2)	C1—C6—C7—N1	7.9 (10)
O3—Zn2—O1—C1	−172.8 (5)	C5—C6—C7—N1	−173.8 (6)
N4—Zn2—O1—C1	−108.4 (5)	N1—N2—C8—N3	172.5 (5)
Cl2—Zn2—O1—C1	89.1 (5)	N1—N2—C8—S1	−6.3 (8)
S2—Zn2—O1—C1	−25.3 (5)	C9—N3—C8—N2	−179.5 (6)
O3—Zn2—O1—Zn1	1.46 (16)	C9—N3—C8—S1	−0.7 (9)
N4—Zn2—O1—Zn1	65.9 (3)	Zn1—S1—C8—N2	10.1 (5)
Cl2—Zn2—O1—Zn1	−96.67 (16)	Zn1—S1—C8—N3	−168.5 (5)
S2—Zn2—O1—Zn1	148.99 (14)	C8—N3—C9—C10	123.0 (7)
O3—Zn1—O1—C1	172.8 (5)	C8—N3—C9—C14	−59.2 (9)
N1—Zn1—O1—C1	37.7 (5)	C14—C9—C10—C11	0.0 (11)
Cl1—Zn1—O1—C1	−70.2 (5)	N3—C9—C10—C11	177.9 (7)
S1—Zn1—O1—C1	91.2 (5)	C9—C10—C11—C12	0.0 (12)
O3—Zn1—O1—Zn2	−1.48 (17)	C10—C11—C12—C13	−0.1 (12)
N1—Zn1—O1—Zn2	−136.6 (2)	C11—C12—C13—C14	0.1 (11)
Cl1—Zn1—O1—Zn2	115.56 (14)	C12—C13—C14—C9	−0.1 (10)
S1—Zn1—O1—Zn2	−83.1 (3)	C10—C9—C14—C13	0.0 (10)
O1—Zn1—O3—C15	172.6 (5)	N3—C9—C14—C13	−177.8 (6)
N1—Zn1—O3—C15	−120.5 (5)	Zn1—O3—C15—C16	−25.5 (8)
Cl1—Zn1—O3—C15	77.4 (5)	Zn2—O3—C15—C16	144.2 (4)
S1—Zn1—O3—C15	−34.5 (5)	Zn1—O3—C15—C20	154.2 (4)
O1—Zn1—O3—Zn2	1.46 (16)	Zn2—O3—C15—C20	−36.1 (7)
N1—Zn1—O3—Zn2	68.3 (3)	O3—C15—C16—C17	179.5 (5)
Cl1—Zn1—O3—Zn2	−93.78 (16)	C20—C15—C16—C17	−0.2 (9)
S1—Zn1—O3—Zn2	154.36 (14)	C15—C16—C17—O4	178.2 (5)
O1—Zn2—O3—C15	−173.5 (5)	C15—C16—C17—C18	−0.5 (9)
N4—Zn2—O3—C15	46.3 (4)	O4—C17—C18—C19	−178.7 (5)
Cl2—Zn2—O3—C15	−57.2 (4)	C16—C17—C18—C19	0.1 (9)
S2—Zn2—O3—C15	108.0 (4)	C17—C18—C19—C20	0.9 (9)
O1—Zn2—O3—Zn1	−1.49 (17)	C18—C19—C20—C15	−1.6 (9)
N4—Zn2—O3—Zn1	−141.7 (2)	C18—C19—C20—C21	−178.4 (6)
Cl2—Zn2—O3—Zn1	114.81 (14)	O3—C15—C20—C19	−178.5 (5)
S2—Zn2—O3—Zn1	−80.0 (2)	C16—C15—C20—C19	1.1 (8)
O3—Zn1—N1—C7	−92.8 (5)	O3—C15—C20—C21	−2.0 (9)
O1—Zn1—N1—C7	−29.0 (5)	C16—C15—C20—C21	177.7 (6)
Cl1—Zn1—N1—C7	70.6 (5)	N5—N4—C21—C20	−179.5 (5)
S1—Zn1—N1—C7	172.7 (5)	Zn2—N4—C21—C20	17.4 (8)
O3—Zn1—N1—N2	101.5 (4)	C19—C20—C21—N4	−172.0 (6)
O1—Zn1—N1—N2	165.4 (4)	C15—C20—C21—N4	11.5 (9)
Cl1—Zn1—N1—N2	−95.0 (4)	N4—N5—C22—N6	176.6 (5)
S1—Zn1—N1—N2	7.1 (4)	N4—N5—C22—S2	−1.8 (8)
C7—N1—N2—C8	−170.3 (5)	C23—N6—C22—N5	−172.2 (5)
Zn1—N1—N2—C8	−3.2 (7)	C23—N6—C22—S2	6.2 (9)
O1—Zn2—N4—C21	−98.7 (5)	Zn2—S2—C22—N5	8.0 (5)
O3—Zn2—N4—C21	−37.0 (5)	Zn2—S2—C22—N6	−170.2 (5)
Cl2—Zn2—N4—C21	65.5 (5)	C22—N6—C23—C28	−41.3 (10)
S2—Zn2—N4—C21	172.1 (5)	C22—N6—C23—C24	139.9 (6)
O1—Zn2—N4—N5	98.5 (4)	C28—C23—C24—C25	0.3 (10)
O3—Zn2—N4—N5	160.2 (4)	N6—C23—C24—C25	179.2 (6)
Cl2—Zn2—N4—N5	−97.3 (4)	C23—C24—C25—C26	0.5 (12)
S2—Zn2—N4—N5	9.3 (3)	C24—C25—C26—C27	0.0 (13)
C21—N4—N5—C22	−171.8 (5)	C25—C26—C27—C28	−1.4 (13)
Zn2—N4—N5—C22	−7.4 (6)	C24—C23—C28—C27	−1.7 (11)
Zn2—O1—C1—C2	−36.4 (8)	N6—C23—C28—C27	179.5 (7)
Zn1—O1—C1—C2	150.7 (4)	C26—C27—C28—C23	2.2 (13)
Zn2—O1—C1—C6	142.5 (5)	C33—N8—C32—O6	−3.4 (11)
Zn1—O1—C1—C6	−30.5 (8)	C34—N8—C32—O6	−177.8 (6)
O1—C1—C2—C3	178.2 (5)	C36—N9—C35—O7	1.1 (10)
C6—C1—C2—C3	−0.7 (9)	C37—N9—C35—O7	178.5 (6)
C1—C2—C3—O2	−178.9 (6)	C39—N10—C38—O8	4.0 (10)
C1—C2—C3—C4	0.1 (9)	C40—N10—C38—O8	−176.6 (6)
O2—C3—C4—C5	180.0 (6)	C30—N7—C29—O5	0.3 (4)
C2—C3—C4—C5	0.9 (10)	C31—N7—C29—O5	−179.6 (4)
C3—C4—C5—C6	−1.4 (10)	C30'—N7'—C29'—O5'	179.9 (4)
O1—C1—C6—C5	−178.7 (5)	C31'—N7'—C29'—O5'	0.0 (4)

### Hydrogen-bond geometry (Å, °)

**Table:**

*D*—H···*A*	*D*—H	H···*A*	*D*···*A*	*D*—H···*A*
O2—H2o···O5	0.84	1.76	2.595 (9)	170
O4—H4o···O6	0.84	1.82	2.657 (6)	175
N2—H2···O7	0.88	1.89	2.713 (6)	156
N3—H3···O7	0.84	2.18	2.940 (6)	151
N5—H5···O8	0.88	1.90	2.717 (6)	154
N6—H6···O8	0.84	2.08	2.841 (6)	150
